# Local Intrapulmonary Hemostatic Therapies for Pediatric Pulmonary Hemorrhage: A Systematic Review and Meta-Analysis

**DOI:** 10.7759/cureus.102056

**Published:** 2026-01-22

**Authors:** Hind A Bafaqih, Huda M Zarie, Adnan B Alkurdi, Abdullah A Qasem, Thageef K Althugafi, Yasser M Al Saheel

**Affiliations:** 1 Pediatric Critical Care, Prince Sultan Military Medical City, Riyadh, SAU; 2 Pediatric Critical Care, King Abdulaziz Hospital, Jeddah, SAU

**Keywords:** diffuse alveolar hemorrhage, intrapulmonary hemostatic therapy, pediatric pulmonary hemorrhage, recombinant activated factor vii, systematic review and meta-analysis, tranexamic acid

## Abstract

Pediatric pulmonary hemorrhage, including diffuse alveolar hemorrhage and severe hemoptysis, is a life-threatening condition associated with high morbidity and mortality. Management is largely supportive, and systemic hemostatic agents may be limited by thrombotic risk. Local intrapulmonary hemostatic therapies, such as tranexamic acid (TXA) and recombinant activated factor VII (rFVIIa), have emerged as targeted strategies to control bleeding while minimizing systemic exposure. However, the available evidence remains fragmented.

We conducted a systematic review and meta-analysis in accordance with PRISMA 2020 guidelines and a prospectively registered PROSPERO protocol (CRD420251273408). PubMed/MEDLINE, Embase, Scopus, Web of Science, and the Cochrane Library were searched from inception to the final search date. Studies including children (0-18 years) with pulmonary hemorrhage treated with local intrapulmonary TXA, rFVIIa, or combination therapy were eligible. Primary outcomes were bleeding control and safety. Random-effects meta-analyses of pooled proportions were performed where feasible.

Six studies comprising 111 pediatric patients met the inclusion criteria, of which five were included in quantitative synthesis. TXA-based local therapy achieved a pooled bleeding control rate of 86.6% (95% CI: 63.9%-95.9%) with moderate heterogeneity, while intrapulmonary rFVIIa achieved a pooled bleeding control rate of 82.5% (95% CI: 62.9%-93.0%) with low heterogeneity. No statistically significant difference was observed between therapies. Recurrence of hemorrhage and transfusion requirements were variably reported but appeared reduced following successful bleeding control. No consistent signal of treatment-related thromboembolic complications was identified, including among oncology/hematopoietic cell transplantation and extracorporeal membrane oxygenation-supported patients.

Local intrapulmonary hemostatic therapies with TXA or rFVIIa are associated with high rates of bleeding control in pediatric pulmonary hemorrhage and appear to be safe adjunctive options in critically ill children. Given the predominance of small observational studies and heterogeneous populations, prospective multicenter studies are needed to define optimal patient selection, dosing strategies, and safety profiles and to clarify the role of these therapies within stepwise management algorithms.

## Introduction and background

Pulmonary hemorrhage in children represents a life-threatening clinical emergency characterized by bleeding into the lower respiratory tract, often manifesting as diffuse alveolar hemorrhage (DAH) or massive hemoptysis [1]. It occurs across a broad spectrum of conditions, including hematologic malignancies, bone marrow or hematopoietic cell transplantation (HCT), congenital heart disease, autoimmune disorders, severe infections, and complications of extracorporeal membrane oxygenation (ECMO) support [2]. Despite advances in pediatric critical care, pulmonary hemorrhage remains associated with substantial morbidity and mortality, largely driven by hypoxemic respiratory failure and the severity of underlying disease [3].

Management of pediatric pulmonary hemorrhage is challenging and primarily supportive, focusing on airway protection, mechanical ventilation, transfusion support, and treatment of the underlying etiology [4]. Systemic hemostatic agents have historically been used; however, their efficacy is variable and their use may be limited by an increased risk of thromboembolic complications, particularly in critically ill children. Consequently, there has been growing interest in local intrapulmonary hemostatic therapies, which aim to achieve targeted control of bleeding while minimizing systemic exposure [5].

Tranexamic acid (TXA), a synthetic antifibrinolytic agent, inhibits plasminogen activation and fibrin degradation. While systemic TXA has an established role in trauma and perioperative bleeding, inhaled or nebulized TXA has emerged as a promising local therapy for pulmonary hemorrhage [6]. Several pediatric and adult studies have demonstrated that inhaled TXA can effectively control hemoptysis with a rapid onset of action and a favorable safety profile, including in critically ill and mechanically ventilated patients [7,8].

Another locally administered hemostatic agent is recombinant activated factor VII (rFVIIa). When delivered intrapulmonary via bronchoscopy or endotracheal tube, rFVIIa promotes localized thrombin generation at sites of alveolar bleeding without significant systemic activation of the coagulation cascade. Case series and cohort studies in pediatric populations, particularly among oncology and transplant patients, have reported high rates of bleeding control using intrapulmonary rFVIIa, even in refractory cases [9,10].

Despite increasing clinical use, the evidence base supporting local intrapulmonary hemostatic therapies in pediatric pulmonary hemorrhage remains fragmented, consisting largely of small observational studies and case series, with variability in patient populations, dosing strategies, and outcome definitions. To date, no comprehensive systematic review and meta-analysis have synthesized the available evidence across different local hemostatic approaches, nor examined their comparative effectiveness and safety.

Importantly, findings from adult studies cannot be directly extrapolated to pediatric pulmonary hemorrhage. Children differ substantially from adults in terms of developmental hemostasis, with age-dependent variations in coagulation factor levels, fibrinolytic activity, and platelet function that may influence both bleeding patterns and responses to hemostatic therapies. In addition, the etiology and clinical context of pulmonary hemorrhage in children, often related to congenital disease, hematologic malignancy, HCT, immune-mediated disorders, or extracorporeal life support, differs markedly from adult populations, where smoking-related lung disease, vasculitis, or cardiovascular conditions predominate. These biological and clinical differences underscore the need for pediatric-specific evidence when evaluating the efficacy and safety of local intrapulmonary hemostatic interventions.

Accordingly, this study aims to systematically review and quantitatively synthesize the available literature on local intrapulmonary hemostatic therapies, including nebulized TXA, intrapulmonary rFVIIa, and their combinations, in the management of pediatric pulmonary hemorrhage. By pooling outcome data where feasible and providing structured narrative synthesis where quantitative analysis is not possible, this review seeks to inform clinical decision-making and identify gaps to guide future research.

## Review

Methods

Study Design

We conducted a systematic review and meta-analysis to evaluate the effectiveness and safety of local intrapulmonary hemostatic therapies for pediatric pulmonary hemorrhage, including nebulized TXA, intrapulmonary/bronchoscopic rFVIIa, and their combinations. The protocol was prospectively registered with PROSPERO (registration number: CRD420251273408), and the conduct and reporting adhered to Preferred Reporting Items for Systematic Reviews and Meta-Analyses (PRISMA) 2020 guidance [11]. Because only published, de-identified data were analyzed, institutional ethics approval was not required.

Search Strategy and Study Selection

A comprehensive, librarian-assisted search was performed in PubMed/MEDLINE, Embase, Scopus, Web of Science, and the Cochrane Library from database inception to the final search date. The strategy combined controlled vocabulary and free-text terms for pulmonary hemorrhage/diffuse alveolar hemorrhage together with terms for tranexamic acid, recombinant factor VIIa, and local intrapulmonary administration, using the Boolean operator “OR” within concepts and “AND” between concepts.

A representative PubMed syntax was: (“Pulmonary Hemorrhage”[Mesh] OR “Diffuse Alveolar Hemorrhage”[Mesh] OR “pulmonary hemorrhage” OR “pulmonary hemorrhage” OR “diffuse alveolar hemorrhage” OR DAH OR hemoptysis OR hemoptysis) AND (tranexamic OR “tranexamic acid” OR TXA OR “Antifibrinolytic Agents”[Mesh] OR “factor VIIa” OR “recombinant activated factor VII” OR rFVIIa) AND (nebulize OR inhaled OR “aerosolized” OR “endobronchial” OR intrapulmonary OR bronchoscopy OR instillation) AND (child OR pediatric OR pediatric OR infant OR neonate OR adolescent).

Equivalent mappings and field tags were adapted for the remaining databases. To enhance completeness, we also searched ClinicalTrials.gov and the WHO International Clinical Trials Registry Platform, screened reference lists of included articles and relevant reviews, and performed citation tracking of key studies.

All retrieved citations were exported to Rayyan.ai, where duplicates were automatically removed. Two reviewers independently screened titles and abstracts against prespecified criteria, followed by full-text assessment in Rayyan.

Inclusion Criteria

Population: Children and adolescents aged 0-18 years with pulmonary hemorrhage, including DAH or clinically significant hemoptysis, managed in pediatric intensive care units, oncology or HCT units, ECMO centers, or pediatric wards.

Intervention: At least one local intrapulmonary hemostatic therapy, defined as: nebulized or inhaled TXA; intrapulmonary or bronchoscopic instillation of TXA; nebulized or inhaled rFVIIa; intrapulmonary or bronchoscopic instillation of rFVIIa; or any combination of these agents.

Comparator: Standard supportive care without local intrapulmonary hemostatic therapy, usual care including systemic hemostatic agents only, alternative local regimens, or before-after comparisons within the same cohort, when reported.

Outcomes: Studies reporting at least one predefined outcome with extractable numerical data, including bleeding control or cessation, recurrence of hemorrhage, transfusion requirements, duration of mechanical ventilation, intensive care unit or hospital length of stay, mortality, or treatment-related adverse events.

Study design: Observational studies, including prospective or retrospective cohort studies and case series involving at least three pediatric patients receiving local intrapulmonary hemostatic therapy. Registered clinical trials were screened to identify eligible observational reports or unpublished results but were not required to be randomized for inclusion.

Exclusion Criteria

Studies were excluded if they involved adult-only cohorts without separable pediatric data, or mixed adult-pediatric populations in which fewer than 80% of participants were younger than 18 years and pediatric-specific results could not be isolated. Studies evaluating systemic TFA or systemic rFVIIa without local intrapulmonary administration were also excluded. In addition, single-patient case reports, narrative reviews, editorials, conference abstracts lacking sufficient extractable data, and animal or in vitro studies were not eligible for inclusion.

For mixed adult-pediatric studies, inclusion was permitted when pediatric data were explicitly reported or when ≥80% of the cohort was within the pediatric age range. Disagreements regarding study eligibility were resolved by discussion or consultation with a third reviewer. Reasons for full-text exclusion were documented and summarized in a PRISMA 2020 flow diagram.

Data Extraction

After confirming study eligibility using Rayyan.ai, two reviewers independently extracted data using a standardized Microsoft Excel form. Extracted variables included the following.

Study characteristics: first author, year of publication, country, study design (observational cohort or case series), clinical setting (pediatric intensive care unit, oncology or transplant unit, ECMO center), and journal.

Patient characteristics: number of pediatric patients receiving local intrapulmonary hemostatic therapy; age (reported as mean or median, with range or standard deviation when available); sex distribution; underlying conditions (hematologic or oncologic disease, hematopoietic stem cell transplantation, autoimmune disease, infection, congenital heart disease, or ECMO support); type of pulmonary hemorrhage (diffuse alveolar hemorrhage or hemoptysis); and baseline respiratory support (invasive ventilation or ECMO use), when reported.

Intervention details: type of local hemostatic therapy (nebulized or inhaled TFA, intrapulmonary or bronchoscopic rFVIIa, or combination regimens); route of administration (nebulization/inhalation, endotracheal tube instillation, or bronchoscopy-guided instillation); dosing regimen, frequency, and duration; timing of therapy relative to hemorrhage onset; and concomitant systemic therapies, when reported.

Outcomes: bleeding control (number of patients achieving complete cessation or clinically meaningful improvement, as defined by each study); recurrence of hemorrhage; transfusion requirements; duration of mechanical ventilation; length of intensive care unit or hospital stay; and mortality, as reported.

Safety outcomes: reported adverse events potentially related to local therapy, including thromboembolic complications and airway-related events.

All extracted data were cross-checked by a second reviewer for accuracy and consistency. Discrepancies were resolved through discussion and consensus. When essential data were missing or unclear, corresponding authors were contacted for clarification. The final dataset was stored in Microsoft Excel and imported into Review Manager (RevMan) version 5.4 for quantitative synthesis.

Risk-of-Bias Assessment

The methodological quality of included studies was independently assessed by two reviewers using the Newcastle-Ottawa Scale (NOS), which is validated for observational studies [12]. The NOS evaluates three domains: selection of study groups, comparability of cohorts, and ascertainment of outcomes, with a maximum score of nine points. Studies were categorized as having low risk of bias (≥7 points), moderate risk of bias (5-6 points), or high risk of bias (<5 points). Any disagreements between reviewers were resolved through discussion and consensus. Risk-of-bias assessments were summarized in tabular form and were considered when interpreting the robustness of pooled estimates and sensitivity analyses.

Statistical Analysis

All quantitative analyses were performed using Review Manager (RevMan) version 5.4. For single-arm studies, the primary outcome (control of pulmonary hemorrhage) was analyzed as a proportion with corresponding 95% confidence intervals (CIs). To stabilize variances, proportions were logit-transformed, and pooled estimates were calculated using the generic inverse variance method under a random-effects model, accounting for anticipated clinical and methodological heterogeneity across studies. Pooled log odds were subsequently back-transformed to proportions for clinical interpretation.

Separate pooled analyses were conducted according to the local hemostatic strategy, including nebulized or inhaled TFA and intrapulmonary or bronchoscopic rFVIIa. Combination local regimens and studies with insufficient data were synthesized narratively and were not included in quantitative pooling.

Between-study heterogeneity was assessed using Cochran’s Q test and quantified with the I² statistic, with values of <25%, 25-50%, and >50% representing low, moderate, and high heterogeneity, respectively. Subgroup analyses were performed by type of local hemostatic therapy, and sensitivity analyses were conducted by excluding studies at high risk of bias to assess the robustness of pooled estimates.

Secondary outcomes, including recurrence of hemorrhage, transfusion requirements, duration of mechanical ventilation, intensive care unit length of stay, mortality, and adverse events, were synthesized narratively due to heterogeneity in reporting and insufficient data for meta-analysis.

Formal assessment of publication bias using funnel plots or statistical tests was not performed, as the number of studies contributing to each analysis was fewer than 10 and the evidence base consisted predominantly of non-comparative observational studies. Forest plots were generated directly in RevMan.

Results

Study Selection

The selection of studies was conducted in accordance with the PRISMA 2020 guidelines. A total of 286 records were identified through electronic database searches, including PubMed/MEDLINE (n = 92), Embase (n = 74), Scopus (n = 58), Web of Science (n = 42), and the Cochrane Library (n = 20). After removal of 84 duplicate records, 202 records remained for title and abstract screening. Of these, 168 records were excluded due to irrelevance to the study question, adult-only populations, absence of pulmonary hemorrhage, or lack of local intrapulmonary hemostatic interventions.

The full texts of 34 articles were retrieved and assessed for eligibility. Following full-text review, 28 articles were excluded for the following reasons: adult-only studies without extractable pediatric data (n = 7), use of systemic but not local hemostatic therapy (n = 6), single-patient case reports (n = 9), conference abstracts without sufficient extractable data (n = 3), and other exclusions not meeting prespecified inclusion criteria (n = 3).

Ultimately, six studies met the inclusion criteria and were included in the systematic review. Of these, five studies provided sufficient data to be included in the quantitative synthesis (meta-analysis). One additional study, involving a very small pediatric cohort receiving combination nebulized TFA and rFVIIa at the end of life, was included only in the narrative synthesis due to insufficient sample size and limited outcome reporting, but was retained to contextualize the clinical use of combination local hemostatic therapy.

**Figure 1:**
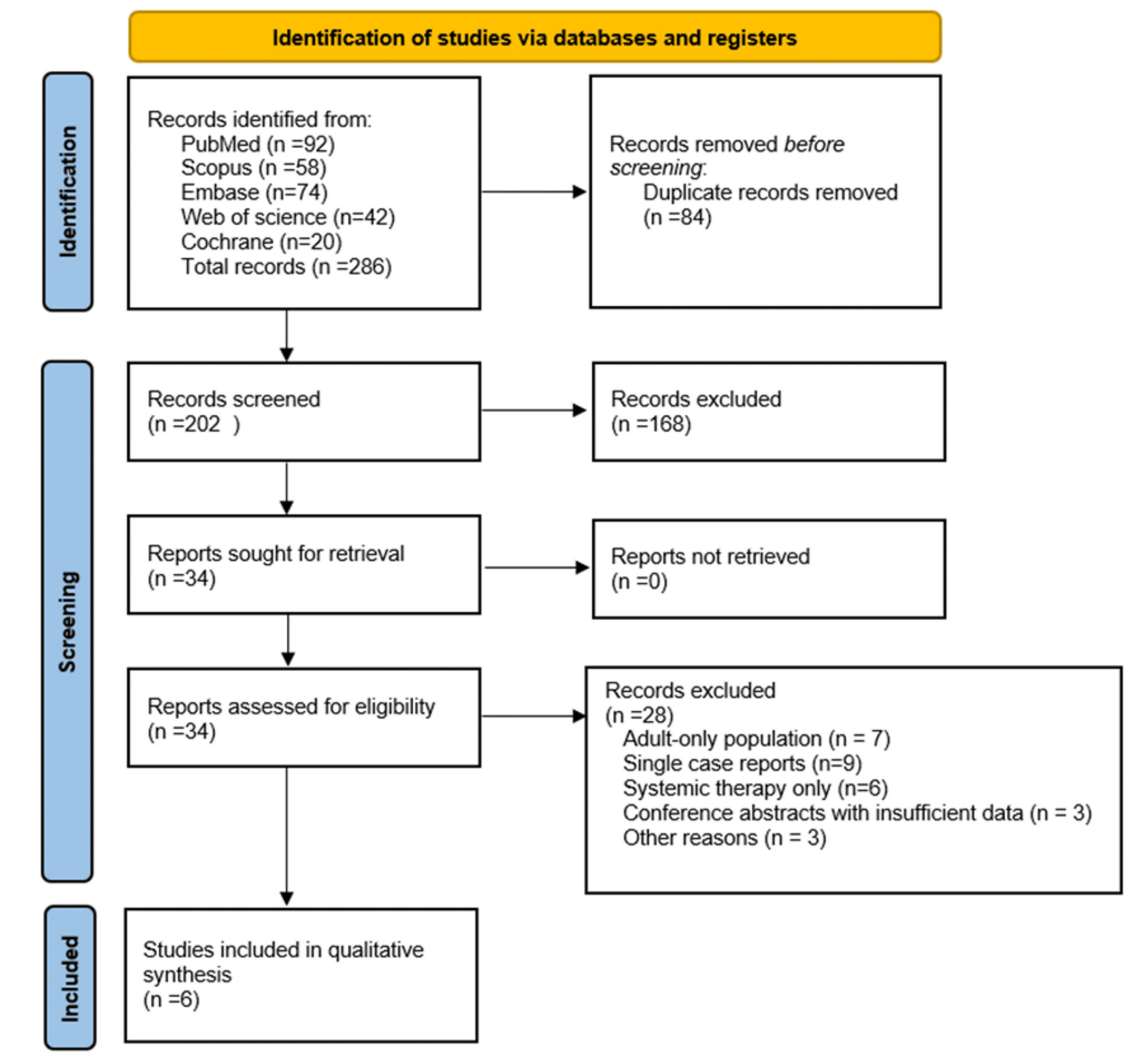
PRISMA 2020 flow diagram of study selection. This figure illustrates the identification, screening, eligibility assessment, and inclusion process for studies evaluating local intrapulmonary hemostatic therapies in pediatric pulmonary hemorrhage. Records were identified through database searches, duplicates were removed, and titles and abstracts were screened for relevance. Full-text articles were assessed for eligibility, with reasons for exclusion documented. Six studies were included in the qualitative synthesis, of which five provided sufficient data for inclusion in the quantitative meta-analysis. PRISMA: Preferred Reporting Items for Systematic Reviews and Meta-Analyses

The study selection process is summarized in the PRISMA flow diagram (Figure 1).

Study Characteristics

A total of six studies published between 2015 and 2025 were included in the systematic review, comprising 111 pediatric patients with pulmonary hemorrhage managed using local intrapulmonary hemostatic therapies (Table 1) [13-18]. Study designs included three retrospective cohort studies, one prospective single-arm pilot study, and two small case series. Most studies were conducted in the United States, one in Saudi Arabia, and one in the Republic of Korea.

**Table 1:** Baseline characteristics of included studies. This table summarizes key characteristics of the included studies, including country of origin, clinical setting, study design, sample size, mean age of participants, underlying conditions, and details of local intrapulmonary hemostatic therapy. Data are presented as reported in the original publications. DAH, diffuse alveolar hemorrhage; ECMO, extracorporeal membrane oxygenation; HCT, hematopoietic cell transplantation; ICU, intensive care unit; PICU, pediatric intensive care unit; rFVIIa, recombinant activated factor VII; TXA, tranexamic acid.

Study ID	Country	Setting	Design	N	Age (Mean +/-SD), months	Population	Local hemostatic regimen
O’Neil, 2020 [13]	USA	Tertiary children’s hospital, PICU	Retrospective cohort	19	85.5 ± 44.0	Critically ill children with pulmonary hemorrhage from mixed etiologies	Inhaled / ETT TXA 250–500 mg per dose, q6–24 h
Bafaqih, 2015 [14]	Saudi Arabia	Tertiary PICU	Prospective single-arm pilot	18	35.4 ± 31.3	Children with intractable diffuse alveolar hemorrhage, often immunocompromised	Step 1: nebulized TXA (250–500 mg); Step 2: nebulized rFVIIa (35–50 µg/kg)
Park, 2015 [15]	Republic of Korea	University children’s hospital, ICU	Retrospective case series	6	129.6 ± 61.2	Children with bronchoscopically confirmed DAH (mainly hematologic/immune disease)	Intrapulmonary rFVIIa via bronchoscopy/ETT, 50 µg/kg per lobe
Hurley, 2024 [16]	USA	Pediatric oncology/HCT ICU	Retrospective cohort	13	151.2 ± 72.0	Oncology/HCT patients with pulmonary hemorrhage or DAH	Intrapulmonary rFVIIa (50–75 µg/kg) via bronchoscopy/ETT
Singleton, 2025 [17]	USA	Pediatric ECMO programs	Retrospective cohort	53	114.7 ± 56.0	Children on ECMO with new pulmonary hemorrhage	Inhaled TXA via ventilator circuit; 250–500 mg per dose
Ganesh, 2025 [18]	USA	Pediatric oncology/palliative care	Small case series	2	102 ± 18	Pediatric cancer patients with hemoptysis at end of life	Combination nebulized TXA + nebulized rFVIIa

The clinical settings were predominantly tertiary pediatric intensive care units (PICUs), including specialized pediatric oncology/HCT ICUs and pediatric ECMO programs. Sample sizes ranged from 2 to 53 patients, with reported mean ages spanning from 35.4 ± 31.3 months in infant-predominant diffuse alveolar hemorrhage cohorts to 151.2 ± 72.0 months in oncology/HCT populations, reflecting substantial heterogeneity in age distribution and underlying disease profiles.

Underlying conditions varied widely across studies. Three studies focused on DAH in children with hematologic or immune-mediated diseases, including malignancy and post-transplant complications [14-16]. One large cohort evaluated children on ECMO who developed new-onset pulmonary hemorrhage [17], while another study included critically ill children with pulmonary hemorrhage from mixed etiologies, such as congenital heart disease, airway bleeding, and other complex medical conditions [13]. One small case series addressed pediatric oncology patients at end of life presenting with hemoptysis in a palliative care context [18].

Local hemostatic interventions included nebulized or endotracheally administered TXA, rFVIIa delivered via bronchoscopy or endotracheal tube, and combination therapy using both agents. TXA dosing typically ranged from 250 to 500 mg per dose, administered at intervals of 6 to 24 hours, while intrapulmonary rFVIIa was generally delivered at doses of 35-75 µg/kg, either as lobar instillation or via endotracheal administration. Combination therapy was reported in two studies [14,18], including one stepwise protocol and one small palliative series.

Risk-of-Bias Assessment

Risk of bias was assessed using the Newcastle-Ottawa Scale (NOS). Overall, study quality ranged from low to high risk of bias. Two retrospective cohort studies [16,17] achieved the highest methodological quality, each with a total NOS score of 8, indicating a low risk of bias. Two additional studies [13,14] demonstrated low-moderate risk of bias (NOS score = 7), primarily due to retrospective or single-arm designs and limited control for confounding. In contrast, two small case series [15,18] were judged to have a high risk of bias (NOS score = 4), reflecting small sample sizes, non-comparative designs, and limited outcome assessment (Table 2). These findings highlight the predominance of observational evidence and support cautious interpretation of pooled results.

**Table 2:** Risk-of-bias assessment of included studies. This table presents the methodological quality assessment of observational studies included in the review. The NOS evaluates studies across three domains: selection of study groups, comparability of cohorts, and outcome assessment. Total scores range from 0 to 9, with higher scores indicating lower risk of bias. Overall risk-of-bias judgments are provided for each study.

Study	Total score	Overall risk of bias
O’Neil, 2020 [13]	7	Low-moderate
Bafaqih, 2015 [14]	7	Low-moderate
Park, 2015 [15]	4	High
Hurley, 2024 [16]	8	Low
Singleton, 2025 [17]	8	Low
Ganesh, 2025 [18]	4	High

Analysis of Bleeding Control by Local Hemostatic Therapy

**Figure 2:**
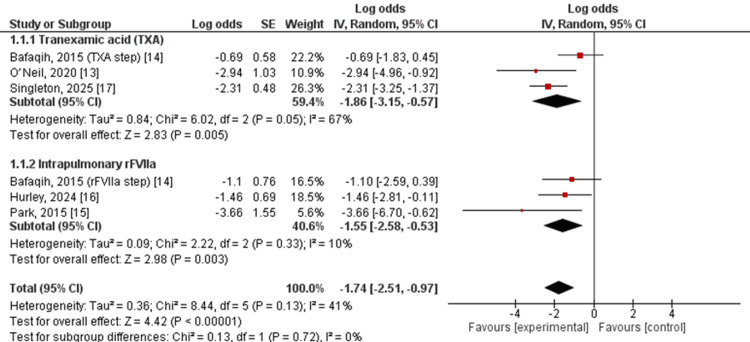
Forest plot of pooled bleeding control rates by local hemostatic strategy This forest plot shows pooled estimates of bleeding control in pediatric pulmonary hemorrhage using a random-effects meta-analysis. Proportions were logit-transformed and analyzed using the generic inverse variance method, then back-transformed for interpretation. Subgroup analyses were performed according to type of local hemostatic therapy. Squares represent individual study estimates, horizontal lines indicate 95% confidence intervals, and diamonds represent pooled estimates.

Subgroup analysis by local hemostatic strategy included TXA-based therapy and intrapulmonary rFVIIa (Figure 2) [13-17]. Among studies evaluating TXA, the pooled log odds of bleeding failure were −1.86 (95% CI: −3.15 to −0.57), corresponding to an estimated bleeding control rate of 86.6% (95% CI: 63.9%-95.9%), with moderate heterogeneity observed across studies (I² = 67%, τ² = 0.84; p = 0.05).

Among studies assessing intrapulmonary rFVIIa, the pooled log odds of bleeding failure were −1.55 (95% CI: −2.58 to −0.53), corresponding to an estimated bleeding control rate of 82.5% (95% CI: 62.9%-93.0%), with low heterogeneity (I² = 10%, τ² = 0.09; p = 0.33).

The test for subgroup differences was not statistically significant (χ² = 0.13, df = 1; p = 0.72), indicating no clear evidence of differential effectiveness between TXA and rFVIIa in achieving control of pediatric pulmonary hemorrhage.

Sensitivity Analysis

A sensitivity analysis was performed excluding the study with a high risk of bias to assess the robustness of the primary findings. After exclusion, the pooled estimates remained consistent with the main analysis. For TFA-based therapy, the pooled log odds of bleeding failure were unchanged (log odds −1.86, 95% CI −3.15 to −0.57), corresponding to an estimated bleeding control rate of approximately 86.6%, with moderate heterogeneity (I² = 67%) [13,14,16,17].

**Figure 3:**
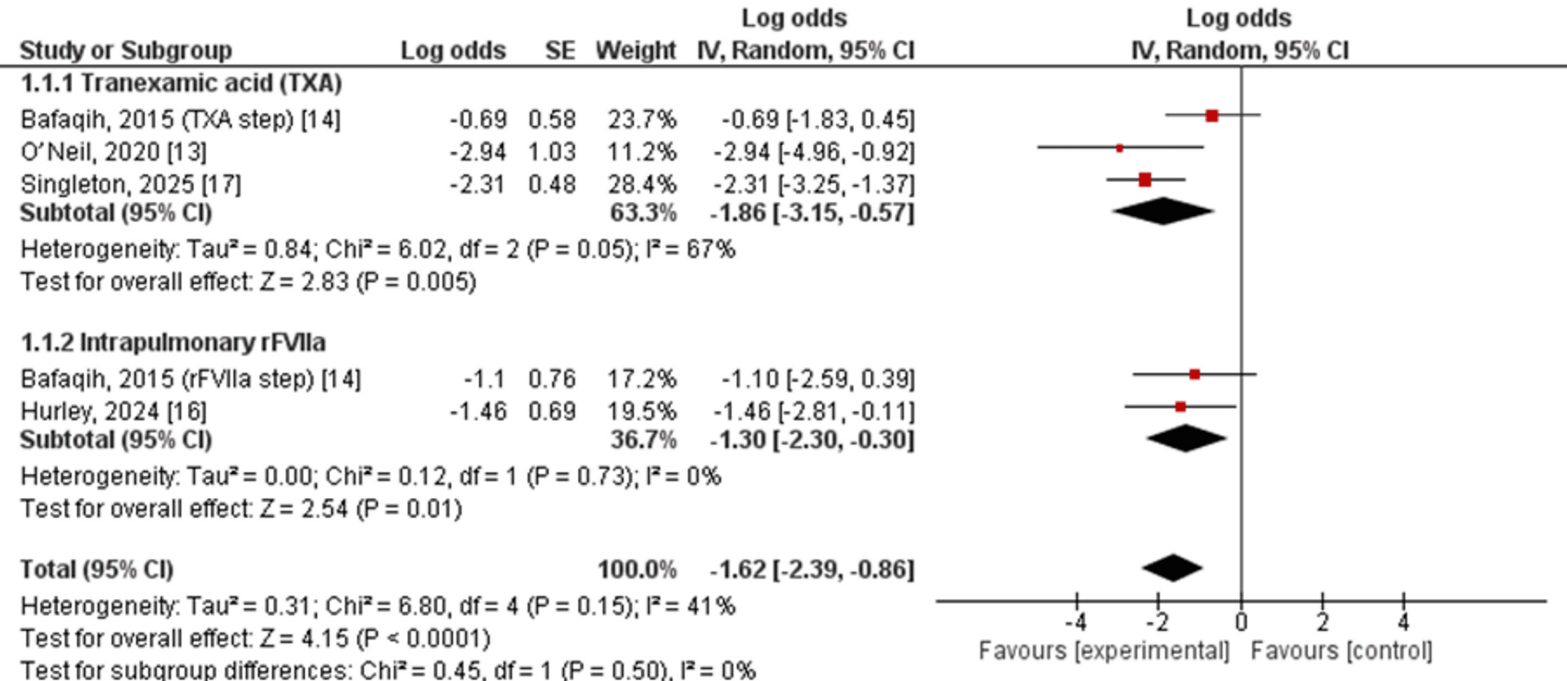
Sensitivity analysis of pooled bleeding control rates excluding high risk-of-bias studies This figure presents the results of a sensitivity analysis evaluating the robustness of pooled bleeding control estimates after exclusion of studies judged to have high risk of bias. The consistency of effect estimates supports the stability of the primary findings across analytical assumptions.

For intrapulmonary rFVIIa, the pooled log odds of bleeding failure were −1.30 (95% CI −2.30 to −0.30), corresponding to an estimated bleeding control rate of approximately 78.6%, with no observed heterogeneity (I² = 0%). The test for subgroup differences remained non-significant (χ² = 0.45, df = 1; p = 0.50), indicating that the overall conclusions were not driven by a single extreme-response or high-risk study (Figure 3).

Secondary Outcomes

Recurrence of pulmonary hemorrhage: Recurrence of pulmonary hemorrhage following initial hemostatic control was inconsistently reported across studies. Among cohorts included in the quantitative synthesis, recurrence after successful local therapy with TFA or intrapulmonary rFVIIa appeared to be uncommon, although definitions and follow-up durations varied substantially.

In a small narrative-only case series involving pediatric oncology patients at end of life treated with combination nebulized TFA and rFVIIa, sustained control of hemoptysis was reported without documented early recurrence, providing supportive qualitative evidence for the feasibility of combination local therapy in selected high-risk contexts [18].

Transfusion requirements: Several studies reported transfusion requirements before and after initiation of local hemostatic therapy. Overall, achievement of bleeding control was associated with a reduction or stabilization in red blood cell and platelet transfusion needs, although reporting thresholds and timeframes were heterogeneous and precluded pooled analysis.

Similarly, in the narrative-only oncology series using combination therapy, control of hemoptysis was accompanied by a reduced need for further transfusion support, suggesting potential benefit in transfusion-dependent patients, albeit based on very limited data.

Mechanical ventilation and intensive care unit length of stay: Data on mechanical ventilation duration and ICU length of stay were reported in a subset of studies included in the meta-analysis. In general, successful control of pulmonary hemorrhage facilitated clinical stabilization, with some patients progressing toward ventilatory weaning. However, these outcomes were strongly influenced by underlying disease severity and comorbid conditions.

In the narrative-only end-of-life cohort, ventilatory and ICU outcomes were not primary endpoints, but symptom control was emphasized as a key goal, underscoring the role of local hemostatic therapy in palliative management rather than survival-oriented endpoints.

Mortality: Mortality was variably reported and largely reflected the severity of underlying illness, including advanced malignancy, post-transplant complications, or critical cardiopulmonary disease. Across studies included in the quantitative synthesis, deaths were generally attributed to the primary disease process rather than uncontrolled pulmonary hemorrhage or adverse effects of local hemostatic therapy.

In the narrative-only oncology series, mortality occurred in the context of terminal illness and was not related to treatment failure, reinforcing that local hemostatic therapy in this setting was primarily used for symptom palliation rather than modification of overall prognosis.

Safety and Adverse Events

Safety outcomes were reported variably across included studies, with no standardized adverse event reporting framework. Overall, local intrapulmonary hemostatic therapies were well tolerated, and no consistent signal of serious treatment-related toxicity was identified.

Among studies evaluating local TXA, no thromboembolic complications attributable to therapy were reported. Respiratory tolerance was generally favorable, and no cases of clinically significant airway obstruction or ventilator circuit compromise related to nebulized or endotracheal administration were described. In patients supported with extracorporeal membrane oxygenation, use of inhaled TFA was not associated with increased circuit thrombosis or oxygenator failure.

For intrapulmonary rFVIIa, no systemic thrombotic events were reported following local administration. Occasional transient airway clot formation was described in isolated cases but did not result in sustained hypoxemia, hemodynamic instability, or need for emergent bronchoscopic intervention. Importantly, no increase in systemic coagulation complications was observed, supporting the localized effect of intrapulmonary delivery.

In the narrative-only series using a combination of TFA and rFVIIa, therapy was administered without reported acute adverse events. Treatment was well tolerated in a palliative care context, with symptom control achieved and no documented therapy-related complications.

Across all studies, adverse outcomes were more commonly related to underlying disease severity rather than local hemostatic therapy itself. However, given the observational design, small sample sizes, and limited duration of follow-up, rare or delayed adverse events cannot be excluded.

Discussion

In this systematic review and meta-analysis of local intrapulmonary hemostatic therapies for pediatric pulmonary hemorrhage, we found that both TXA-based regimens and intrapulmonary rFVIIa were associated with high rates of bleeding control. In pooled single-arm analyses, TXA-based therapy achieved an estimated bleeding control rate of 86.6% (95% CI: 63.9%-95.9%), while intrapulmonary rFVIIa achieved 82.5% (95% CI: 62.9%-93.0%), and there was no statistically significant subgroup difference between strategies. These findings are clinically important because pediatric pulmonary hemorrhage, particularly DAH, often occurs in high-acuity contexts (oncology/HCT, ECMO, severe systemic illness) and is associated with substantial mortality and resource utilization [1-4,19].

The overall signal of benefit for inhaled/local TXA in our pediatric synthesis is directionally consistent with adult data showing that inhaled TXA can shorten time to bleeding cessation and reduce recurrence, including a randomized controlled trial of nebulized TXA in hemoptysis [6] and more recent controlled evidence comparing nebulized versus intravenous administration [7], as well as meta-analytic summaries supporting TXA’s effect on hemoptysis outcomes [8]. In pediatrics, the included cohorts suggest that TXA delivered via nebulization or endotracheal route may serve as a pragmatic, rapidly deployable adjunct to supportive care, including in mechanically ventilated patients and in ECMO settings [20,21]. However, the moderate heterogeneity observed in the TXA subgroup likely reflects clinically meaningful differences in underlying etiologies (DAH vs non-DAH bleeding), acuity, ventilation/ECMO status, dosing intervals, and the operational definition of “bleeding control” across studies [22,23].

For intrapulmonary rFVIIa, our findings align with the mechanistic rationale and prior literature describing effective localized hemostasis when rFVIIa is delivered directly to the alveolar space, potentially minimizing systemic prothrombotic exposure [9,10,24]. Historical reports in DAH (primarily adult or mixed populations) demonstrated feasibility and rapid hemostatic response with pulmonary administration [25], and post-transplant DAH case experience also supports its use in refractory hemorrhage [26]. Importantly, pediatric evidence remains largely observational, but the higher-quality pediatric oncology/HCT ICU cohort contributes meaningful real-world support for intrapulmonary rFVIIa in severe hemorrhage contexts [21].

A key clinical consideration is that pediatric pulmonary hemorrhage is not a single entity; the prognosis and therapeutic goals differ markedly across populations. DAH in transplant and hematologic malignancy settings is often part of complex inflammatory and endothelial injury syndromes and remains associated with poor outcomes despite supportive care [19,22,26]. In such patients, local hemostatic therapy may be most appropriately viewed as a bridge, aimed at stabilizing oxygenation, limiting transfusion escalation, and enabling time for etiologic therapy rather than as a definitive intervention that alters the trajectory of the underlying disease [24,26]. Conversely, in focal or airway-source bleeding (non-DAH hemoptysis), local pharmacologic hemostasis may complement bronchoscopic measures and, when necessary, procedural interventions. Although data are limited, pediatric bronchial artery embolization is recognized as an option for selected cases of significant hemoptysis, reinforcing the broader paradigm of targeted local control strategies [26].

Combination local therapy (stepwise nebulized TXA followed by nebulized rFVIIa, or concurrent use) was reported in limited pediatric experience and generally in highly selected or palliative contexts [22,25]. While these reports support feasibility, they are too sparse to support comparative conclusions. The escalation logic is clinically intuitive, starting with TXA as a lower-complexity local antifibrinolytic, then considering intrapulmonary rFVIIa for refractory bleeding, but prospective protocols and standardized endpoints are needed before recommending routine stepwise algorithms.

Across included studies, no consistent signal of therapy-attributable thromboembolic harm was identified, including among ECMO-supported children receiving inhaled TXA and among oncology/HCT patients receiving intrapulmonary rFVIIa [20,21,23]. This is reassuring, particularly given concerns about systemic antifibrinolytics or procoagulants in critically ill populations. Nonetheless, safety inference is limited by small sample sizes, non-standardized adverse-event capture, and short follow-up windows. This limitation is especially relevant in transplant and oncology cohorts where baseline risks of thrombosis, endothelial injury, and multi-organ failure are substantial [19,22,26]. Therefore, local administration should not be interpreted as risk-free; rather, it should be considered risk-mitigating relative to systemic exposure, with ongoing vigilance for airway obstruction/clot burden, circuit complications on ECMO, and patient-specific thrombotic risk factors.

The evidence base remains constrained by a small number of studies, predominance of observational designs and small case series, heterogeneity in definitions of pulmonary hemorrhage/DAH and “bleeding control,” variable dosing strategies and routes, and confounding by indication in the sickest patients. These factors limit causal inference and restrict our ability to identify optimal agent, dose, timing, and duration. Future research priorities include multicenter prospective registries with standardized definitions (DAH vs non-DAH, severity grading), uniform bleeding-control endpoints and time-to-event reporting, structured adverse-event surveillance (including thrombosis and airway complications), and pragmatic comparative designs in key subgroups (oncology/HCT, ECMO, and mechanically ventilated PICU patients). Given feasibility constraints for large pediatric RCTs, high-quality prospective comparative cohorts may be the most realistic near-term pathway.

## Conclusions

This systematic review and meta-analysis suggest that local intrapulmonary hemostatic therapies, including TXA and rFVIIa, are associated with high rates of bleeding control in pediatric pulmonary hemorrhage without a clear difference in effectiveness between agents. These therapies appear to offer a valuable adjunctive option for rapid stabilization in critically ill children, particularly in high-acuity settings such as oncology or HCT and extracorporeal life support, where systemic therapies may be limited by bleeding or thrombotic risk. Importantly, current evidence supports their use as adjuncts to, rather than replacements for, standard supportive and etiologic management of pulmonary hemorrhage. However, the current evidence is derived predominantly from small observational studies with heterogeneous populations, dosing strategies, and outcome definitions, which limits causal inference and generalizability. Prospective multicenter studies using standardized definitions and clinically meaningful endpoints are needed to better define optimal patient selection, timing, dosing, and safety profiles, and to clarify the role of local hemostatic therapy within stepwise management algorithms for pediatric pulmonary hemorrhage.
